# Targeting lysyl oxidase reduces peritoneal fibrosis

**DOI:** 10.1371/journal.pone.0183013

**Published:** 2017-08-11

**Authors:** Christopher R. Harlow, Xuan Wu, Marielle van Deemter, Fiona Gardiner, Craig Poland, Rebecca Green, Sana Sarvi, Pamela Brown, Karl E. Kadler, Yinhui Lu, J. Ian Mason, Hilary O. D. Critchley, Stephen G. Hillier

**Affiliations:** 1 MRC/University of Edinburgh Centre for Reproductive Health, Edinburgh Medical School, Queen’s Medical Research Institute, 47 Little France Crescent, Edinburgh, United Kingdom; 2 MRC/University of Edinburgh Centre for Inflammation Research, Edinburgh Medical School, Queen’s Medical Research Institute, 47 Little France Crescent, Edinburgh, United Kingdom; 3 University of Manchester, Wellcome Trust Centre for Cell-Matrix Research, Faculty of Life Sciences, Michael Smith Building, Manchester, United Kingdom; Medical University of South Carolina, UNITED STATES

## Abstract

**Background:**

Abdominal surgery and disease cause persistent abdominal adhesions, pelvic pain, infertility and occasionally, bowel obstruction. Current treatments are ineffective and the aetiology is unclear, although excessive collagen deposition is a consistent feature. Lysyl oxidase (Lox) is a key enzyme required for crosslinking and deposition of insoluble collagen, so we investigated whether targeting Lox might be an approach to reduce abdominal adhesions.

**Methods:**

Female C57Bl/6 mice were treated intraperitoneally with multiwalled carbon nanotubes (NT) to induce fibrosis, together with chemical (ß-aminoproprionitrile–BAPN) or miRNA *Lox* inhibitors, progesterone or dexamethasone. Fibrotic lesions on the diaphragm, and expression of fibrosis-related genes in abdominal wall peritoneal mesothelial cells (PMC) were measured. Effects of BAPN and dexamethasone on collagen fibre alignment were observed by TEM. Isolated PMC were cultured with interleukin-1 alpha (IL-1α) and progesterone to determine effects on *Lox* mRNA *in vitro*.

**Results:**

NT-induced fibrosis and collagen deposition on the diaphragm was ameliorated by BAPN, *Lox* miRNA, or steroids. BAPN and dexamethasone disrupted collagen fibres. NT increased PMC *Lox*, C*ol1a1*, *Col3a1* and *Bmp1* mRNA, which was inhibited by steroids. Progesterone significantly inhibited IL-1α induced *Lox* expression by PMC *in vitro*.

**Conclusion:**

Our results provide proof-of-concept that targeting peritoneal Lox could be an effective approach in ameliorating fibrosis and adhesion development.

## Introduction

Aberrant peritoneal fibrosis leading to adhesion formation is a feature of abdominal surgery, infection or disease, resulting in abdominal pain, infertility and, in severe cases, bowel obstruction. Adhesions are also a common feature of endometriosis [1]. Recent reviews [2,3] summarized a range of anti-adhesive barrier and pharmacological measures that have been used to prevent abdominal adhesions. No single adhesion-preventative measure has emerged as unequivocally effective, although randomized controlled trials provide supporting evidence for the use of most barrier/gel agents, and post-operative corticosteroids remain the only likely pharmacological contender [2–4], but even so, the effectiveness of glucocorticoids is controversial [3,5,6]. Fibrosis is a prominent feature of the peritoneum in dialysis patients, and glucocorticoids have been implicated as a potential treatment to reduce fibrosis and the onset of the life-threatening condition of encapsulating peritoneal sclerosis [7].

The precise aetiology of adhesion formation is unclear, but is likely due to excessive collagen deposition resulting from incomplete fibrinolysis at the site of injury/damage to the peritoneal mesothelial cells (PMC) [8]. A key step in collagen deposition requires cross-linking of collagen to form an insoluble matrix, controlled by the copper dependent amine oxidase, lysyl oxidase (Lox) [9]. Lox has been implicated in mouse models of dermal wound healing [10] and scarring [11] as well as fibrosis [12]. *LOX* mRNA increased in rabbit abdominal wall scars [13], and over 30 years ago the lathryogen ß-aminopropionitrile (BAPN), which irreversibly inhibits LOX activity, was found to inhibit skin collagen polymerisation and scarring in rats [14].

Regulation of *LOX* mRNA and enzyme activity has been noted in rat ovarian granulosa cells [15] and also in human ovarian surface epithelial cells (OSE) [16]. IL-1α enhanced and cortisol inhibited *LOX* mRNA expression in human OSE cells [16]. *Lox* mRNA also increased in the parietal peritoneum and PMC of a chlorhexidine gluconate-induced peritoneal fibrosis mouse model [17]. Upregulation of LOX has also recently been implicated in abnormal endometrial function and in proliferation, migration and invasion of endometriotic lesions [18]

In this study, we chose a mouse model of carbon nanotube (NT)-induced fibrosis on the abdominal surface of the diaphragm [19] to investigate the role of Lox in mediating the fibrotic response. We showed that NT-induced fibrosis was accompanied by increased *lox* expression in PMC, and that chemical or miRNA mediated inhibition of *Lox* reduced the fibrotic response. Additionally, we assessed if glucocorticoid and/or progesterone was able to ameliorate the fibrotic response, with the aim of re-examining the role of glucocorticoids and sex steroids, and exploring the mechanism of local steroid action in fibrosis and adhesion formation in the peritoneal cavity. To study the effects of inflammatory and anti-inflammatory factors on the expression of fibrosis-related genes, we collected PMCs from the abdominal wall to determine mRNA expression, and also measured mRNA expression after culturing abdominal wall PMCs *in vitro* in the presence of inflammatory and anti-inflammatory factors.

We propose that inhibition of Lox in abdominal PMC may help reduce inflammation-associated fibrosis and scarring, with implications for the prevention of adhesions following surgery, infection and disease.

## Materials and methods

### Animals

C57Bl/6 female mice were obtained from Harlan, housed under 12 h light: 12 h dark conditions and given standard rodent chow and water *ad libitum*. All animal experiments were conducted under a project license approved by the UK Home Office in accordance with the Animals (Scientific Procedures) Act 1986. Animals used were aged 10–12 weeks at the start of the experiment. A minimum of 3 and a maximum of 9 mice were used in each experimental group. Animals were housed in groups, and randomly allocated to each experimental group. All animals were checked for body condition and overall health on a daily basis by the experimenter.

### Carbon nanotubes (NT)

Multiwalled NT (NT_long2_, [19] were obtained from Dr Matthew Boyles, University of Cambridge. NT were prepared for injection by mixing at a concentration of 1 mg/ml with PBS containing 0.5% BSA (PBS-BSA) (Sigma, Poole, Dorset). The suspension was vortexed for 2 minutes followed by sonication in a waterbath containing ice for 15 minutes. This treatment was repeated for 6 hours after which the suspended NT were stored overnight at 4°C. Prior to use the nanotubes were vortexed and sonicated a further 3 times and then diluted with PBS-BSA to a concentration of 100 μg/ml. Treated mice were injected i.p. with 0.25 ml containing 25 μg NT. Control mice were treated with 0.25 ml PBS-BSA.

### BAPN and steroid treatment

The following treatments were given daily starting on the day of NT treatment and continued for 7 days, unless time was a variable. BAPN (fumarate salt, Sigma) was freshly prepared by dissolving in PBS-BSA at a concentration of 250 mg/ml. Treated mice were injected i.p with 0.2 ml containing 50 mg BAPN (~ 1 g/kg). Progesterone (Sigma) was prepared as a stock solution in 100% ethanol at 10 mg/ml. Aqueous suspension was prepared from this stock at a concentration of 1 mg/ml. Treated mice were injected i.p with 0.2 ml containing 0.2 mg progesterone (~10 mg/kg). Dexamethasone (Sigma) was prepared as a stock solution in 100% ethanol at 10 mg/ml. Aqueous suspension was prepared from this stock at a concentration of 0.1 mg/ml. Treated mice were injected i.p with 0.2 ml containing 0.02 mg dexamethasone (~ 1 mg/kg).

### PMC culture

Mesothelial cells were collected and cultured as described previously [20]. Further details are given in the Supplementary methods (S1 File).

### Molecular cloning and lox miRNA-Lentiviral miRNA constructs

Block-It RNAi system (Invitrogen, Life Technologies, Paisley, Renfrewshire) was used to deliver 21 bp RNAi designed to be specific to mouse LOX variants 1, 2 and 3, which were first incorporated within a pre-miR based on mouse miR-155 and contained within pcDNA6.2-GW-emGFP-miR and screened (S1 Fig and S1 Table). The miR-155 creates a stem loop structure facilitating processing of the RNAi, but specificity is retained to the mRNA target sequences. In addition, two sequences were identified L-225 and -227 and used in all further experiments by first shuttling into pLenti6.2-cppt-CMV-DEST to create pLenti6.2-cppt-CMV-emGFP-L-225 and -227 and then packaged, using methodology detailed in [21]. Large scale packaging, titering and production of serum free lentivirus is described previously [22].

Mock transfected, scrambled sequence transfected, and the two most *in vitro* effective *lox* miRNA constructs (225 and 227) (S1 Fig) were prepared in OPTI-MEM medium (Gibco, Life Technologies, Paisley, Renfrewshire) containing 0.1% polybrene (Sigma). Lentiviral constructs were used at a dose of 7.0 x 10^7^ TU/injection in 0.5 ml OPTI-MEM. Mice were injected with vehicle or vehicle containing miRNA. Two days later animals received a single injection of 25 μg NT (in 0.25 ml PBS/BSA), with one group receiving vehicle alone.

### Sample collection—Abdominal wall mesothelial cells

Seven days after NT injection (unless time was a variable), and 24 h after the final vehicle, BAPN and/or steroid injection, animals were killed by exposure to increasing CO_2_ concentrations followed by cervical dislocation. Abdominal wall peritoneal mesothelial cells were collected by removing the skin and pinning out the lateral abdominal wall between the hindlimb and ribcage onto clean foil (S2 Fig). A 1 cm tall section cut from the top of sterile 50 ml Falcon tube (VWR, Lutterworth, Leicestershire, UK) was placed on the exposed mesothelial surface and held down firmly. 0.7ml RNA lysis buffer (RNEasy, Qiagen) was placed inside the ring and the mesothelial surface was scraped for 10–15 seconds using a 1.8 cm wide Costar® cell scraper (Corning). The resulting lysate was removed by pipette and stored at -80°C until required for RNA extraction. Evidence that this method removed only the mesothelial cells was obtained by observing cytokeratin staining of tissue that had and had not undergone this treatment (S3 Fig)

### RNA extraction, reverse transcription and quantitative real-time PCR (qRT–PCR)

RNA was extracted from mesothelial cell lysates using the RNEasy micro extraction kit, with on column DNAse digestion (Qiagen), following the manufacturer’s instructions. RNA (200 ng) was reverse-transcribed using a High Capacity cDNA Reverse Transcription Kit (SuperScript® VILO cDNA Synthesis Kit, Life Technologies), following the manufacturer’s protocol. Quantification of total transcripts was performed using TaqMan® Gene Expression Assay (S2 Table) and 18S ribosomal RNA was used for normalization (Life Technologies). qRT–PCR was performed using the ABI Prism 7600 Sequence Detection System, using TaqMan® Universal PCR Master Mix (Life Technologies). Expression was displayed as fold change relative to the expression level in control animals or 12 h control cell cultures. Primer/probe sets were pre-validated assay-on-demand reagents (Qiagen). The assay probes all crossed exon boundaries. Details of the probes are provided (S2 Table).

### Sample collection—Diaphragms

The diaphragms were removed complete with surrounding ribcage (to preserve the tissue architecture), as described in detail previously [19]. The section of intact ribcage was fixed for 16–24 h using neutral buffered formalin (NBF).

### Sample collection—Abdominal wall

A section of abdominal wall (approximately 1cm^2^) was removed and pinned out with the mesothelial surface uppermost on a polystyrene block submerged under NBF, where it was fixed for 16–24 h. This method preserved the structure of the abdominal wall and prevented it from curling up into a tube.

### Histology

Once fixed, a horizontal section of the diaphragm measuring approximately 20 x 3 mm (incorporating both the muscular and tendonous regions of the diaphragm) was cut out using a scalpel blade and embedded on edge in paraffin wax. The abdominal wall tissue was similarly embedded on edge. Paraffin embedded sections (5 μm thick) were dewaxed and stained for collagen using picrosirius red. The stained sections were tiled to give a complete representation of the diaphragm section using Media Cybernetics image pro plus software with Stage Pro. The fibrotic lesion area (mm^2^) and lesion area stained for collagen per linear mm of diaphragm were calculated using Adobe Photoshop CS5.1 software.

For details of tissue staining and immunohistochemistry see S1 File.

### Electron microscopy

Samples measuring approximately 3 mm x 2 mm (see above) were cut out from the diaphragm and incubated in glutaraldehyde fixative (2% in 100 mM phosphate buffer, pH 7) for 30 min at 21°C. Samples were transferred to fresh fixative for 2 hours at 4°C, and then in fresh glutaraldehyde fixative (1% in 50 mM phosphate buffer, pH 6.2) supplemented with 1% osmium tetroxide for 40 min at 4°C. Samples were washed in 6 changes of water and transferred to 1% uranyl acetate for 16 hours at 4°C. Dehydration was in graded ethanol and embedding in Agar LV as previously described [23]. Semi-thin (2 μm) sections were placed on a glass slide and stained with 1% toluidine blue, rinsed in water, and dried on a hot plate. Light microscopy was used to identify the regions of interest and for tissue orientation. Ultra-thin sections (70 nm) for transmission electron microscopy were collected using a Leica ultramicrotome and placed on 200 mesh copper grids. Images were taken using a Tecnai 12 BioTwin electron microscope operated at 100 kV accelerating voltage.

### Statistical analysis

Data are presented throughout as mean ± standard error of the mean (SEM). Statistical analysis was performed using Graphpad Prism 5 software. Comparisons between groups was by one- or two-way ANOVA, followed by Tukey’s or Benferroni post-hoc testing respectively. A value of p<0.05 was considered significant for all analyses.

## Results

### Chemical inhibition of LOX reduces NT-induced fibrosis and collagen deposition on the diaphragm

**Fig 1:**
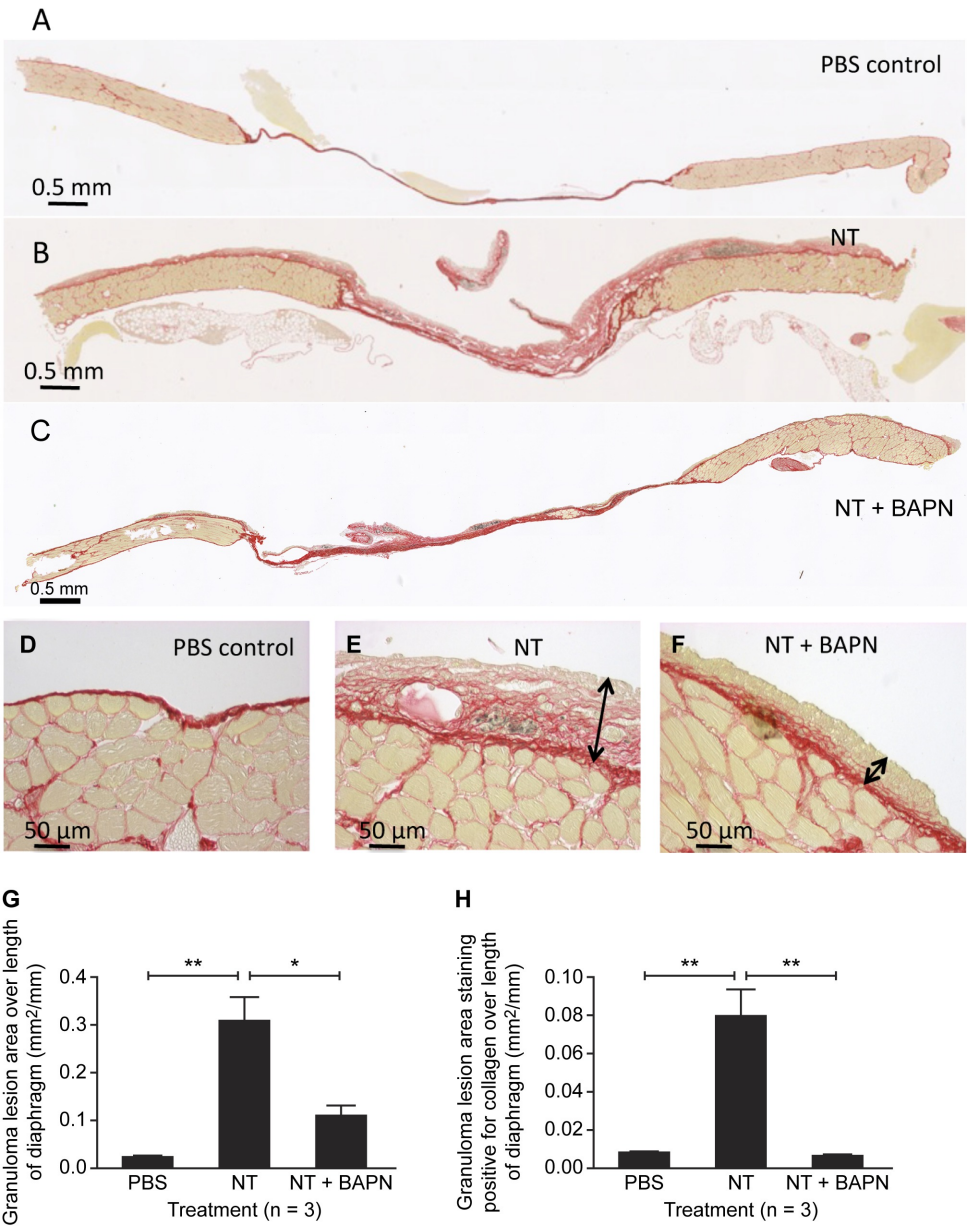
BAPN reduces NT-induced fibrosis on the diaphragm. Effect of i.p. treatment with carbon nanotubes (NT, 25 μg) followed by daily injections for 7 days of the lox inhibitor ß-aminoproprionitrile (BAPN, 1 g/kg) on fibrosis and collagen deposition on the diaphragm. Picrosirius Red stained sections of control diaphragm treated with PBS (A,D), NT (B,E) and NT+BAPN (C,F). Arrows indicate the thickness of the fibrotic lesion. Red staining indicates collagen. Fibrotic granuloma lesion area (G) and extent of collagen staining within the lesion (H) were quantified. Results are expressed as mean ± SEM, n = 3. *, p<0.05; **, p<0.01. One-way ANOVA with Tukey’s multiple comparison post-hoc testing.

Intra-peritoneal injection of NT caused massive accumulation of collagen-rich fibrotic granuloma lesions on the abdominal surface of the diaphragm after 7 days, observed using picrosirius red (PSR) staining (Fig 1A, 1B, 1D and 1E). The presence of accumulated NT fibres was noted (Fig 1E). The granuloma lesions appeared to be on the peritoneal surface, and were associated with an accumulation of macrophages and B lymphocytes (S4 Fig). The thickness of the granuloma lesions and the extent of collagen staining were reduced after 7 days treatment with BAPN (Fig 1C and 1F). Quantification of granuloma lesion area (Fig 1G) and granuloma lesion area staining positive for collagen (Fig 1H) showed that BAPN treatment reduced the effect of NT on both endpoints by 90% (p<0.01)

### BAPN causes ultrastructural changes in collagen fibre alignment in the diaphragm

**Fig 2:**
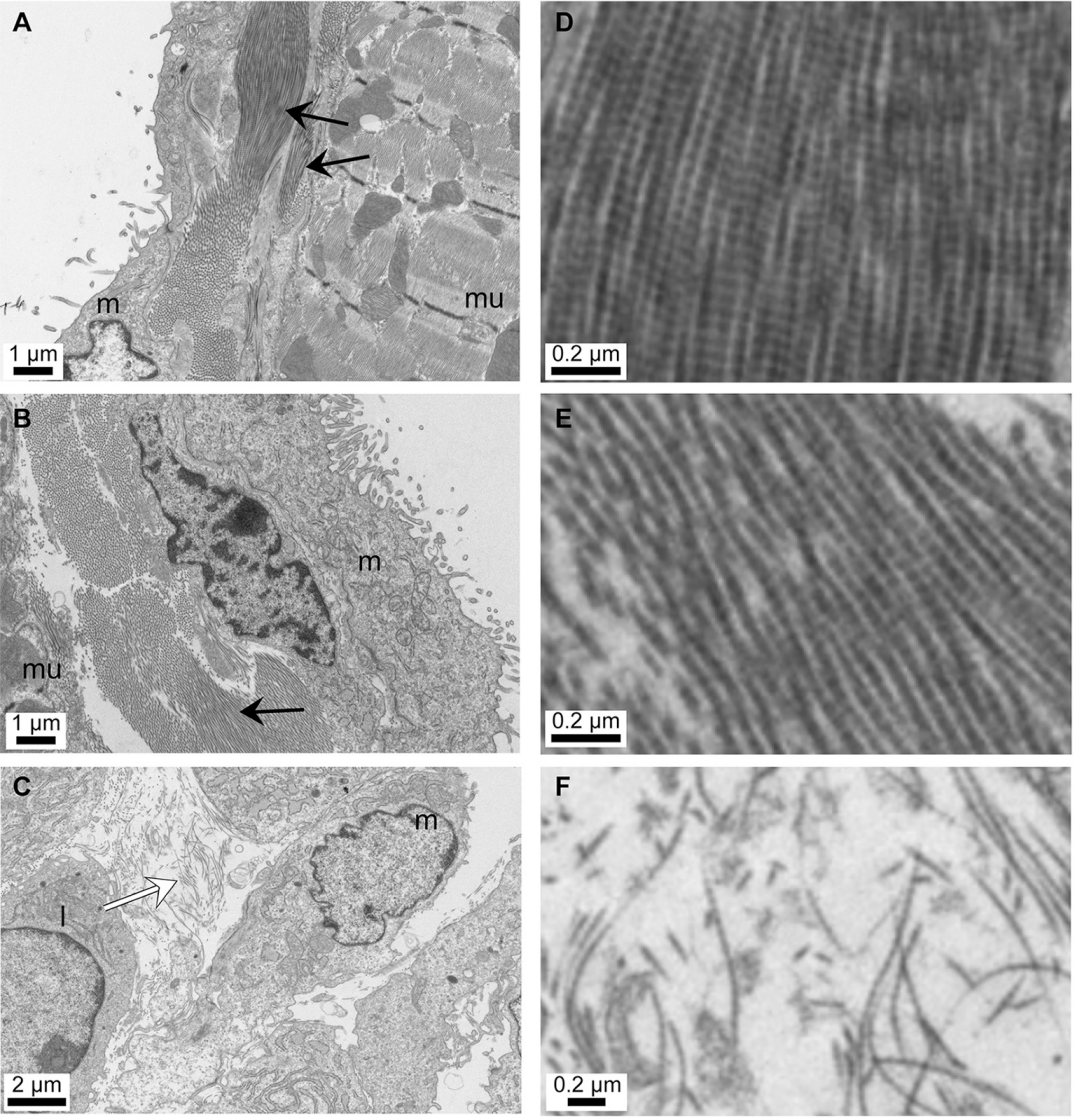
Electron micrographs of mouse diaphragm showing effect of NT and BAPN on sub-epithelial collagen deposition. A,D PBS control; B,E 7 days after NT treatment; C,F 7 days after NT plus daily β-aminopropionitrile (BAPN) treatment. m: mesothelial cells, mu: muscle fibres, l: probable leukocyte. ↑ dense, ordered collagen fibre bundles, ⇧ loose, disordered collagen fibres.

Transmission electron microscopy revealed ordered collagen fibre bundles lying between the peritoneal mesothelial cell layer and the muscle layers (Fig 2A and 2D). Note the presence of typical collagen fibril D-banding striations, with periodicity of 67nm (Fig 2D). NT treatment increased the amount of collagen present (Fig 2B and 2E). BAPN treatment for 7 days after NT caused the collagen fibre bundles to lose their ordered arrangement (Fig 2C and 2F).

### Effect of progesterone and dexamethasone on NT-induced fibrosis and collagen deposition on the diaphragm

**Fig 3:**
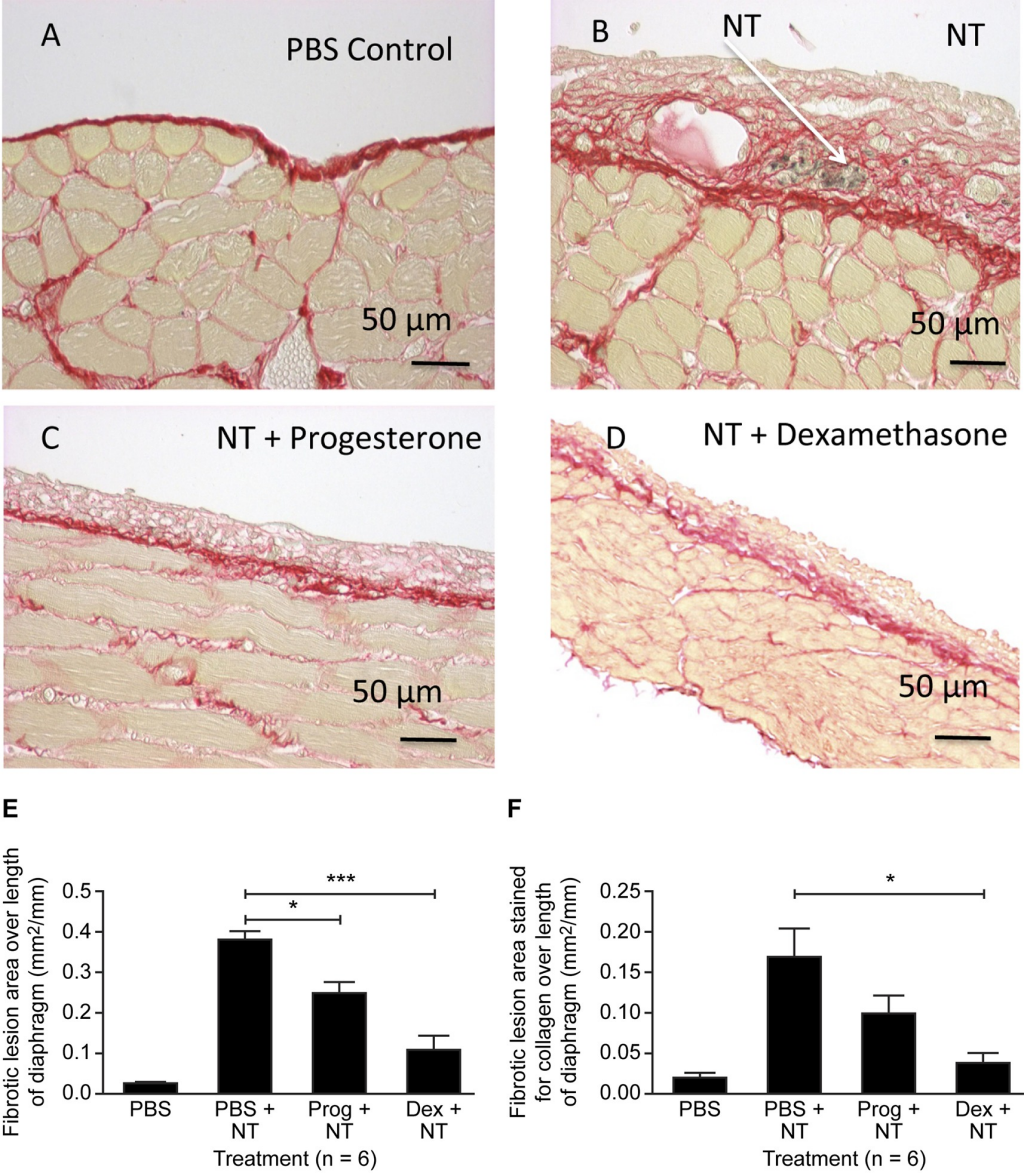
Progesterone and dexamethasone reduce NT-induced fibrosis on the diaphragm. Effect of i.p. treatment with carbon nanotubes (NT, 25 μg) followed by 7 daily injections of PBS (vehicle), progesterone (10 μg) or dexamethasone (1 μg) on fibrosis and collagen deposition on the diaphragm. Picrosirius Red stained sections of control diaphragm treated with PBS (A), NT (B), NT + progesterone (C) and NT + dexamethasone (D). Red staining indicates collagen. Fibrotic granuloma lesion area (E) and extent of collagen staining within the lesion (F) were quantified. Results are expressed as mean ± SEM, n = 6. *, p<0.05; ***, p<0.001. One-way ANOVA with Tukey’s multiple comparison post-hoc testing.

Having established that inhibition of Lox activity with BAPN ameliorated the effects of NT on the diaphragm, we examined if known anti-inflammatory steroids, progesterone and dexamethasone were also able to reduce the effects of NT on the diaphragm. We first determined that the diaphragm mesothelial cells expressed progesterone and glucocorticoid receptor by immunohistochemistry (S5 Fig). PSR staining revealed that the fibrotic granuloma lesion thickness and collagen content induced by NT injection (Fig 3A and 3B) were reduced by both progesterone (Fig 3C) and dexamethasone (Fig 3D) treatment for 7 days. Quantification revealed that granuloma lesion area (Fig 3E) was significantly reduced by both progesterone (p<0.05) and dexamethasone (p<0.01). Granuloma lesion area staining positive for collagen (Fig 3F) was also reduced by progesterone and dexamethasone, although only the effect of dexamethasone was statistically significant (p<0.05).

### Time dependent effects of NT, progesterone and dexamethasone on abdominal wall PMC fibrotic gene expression

**Fig 4:**
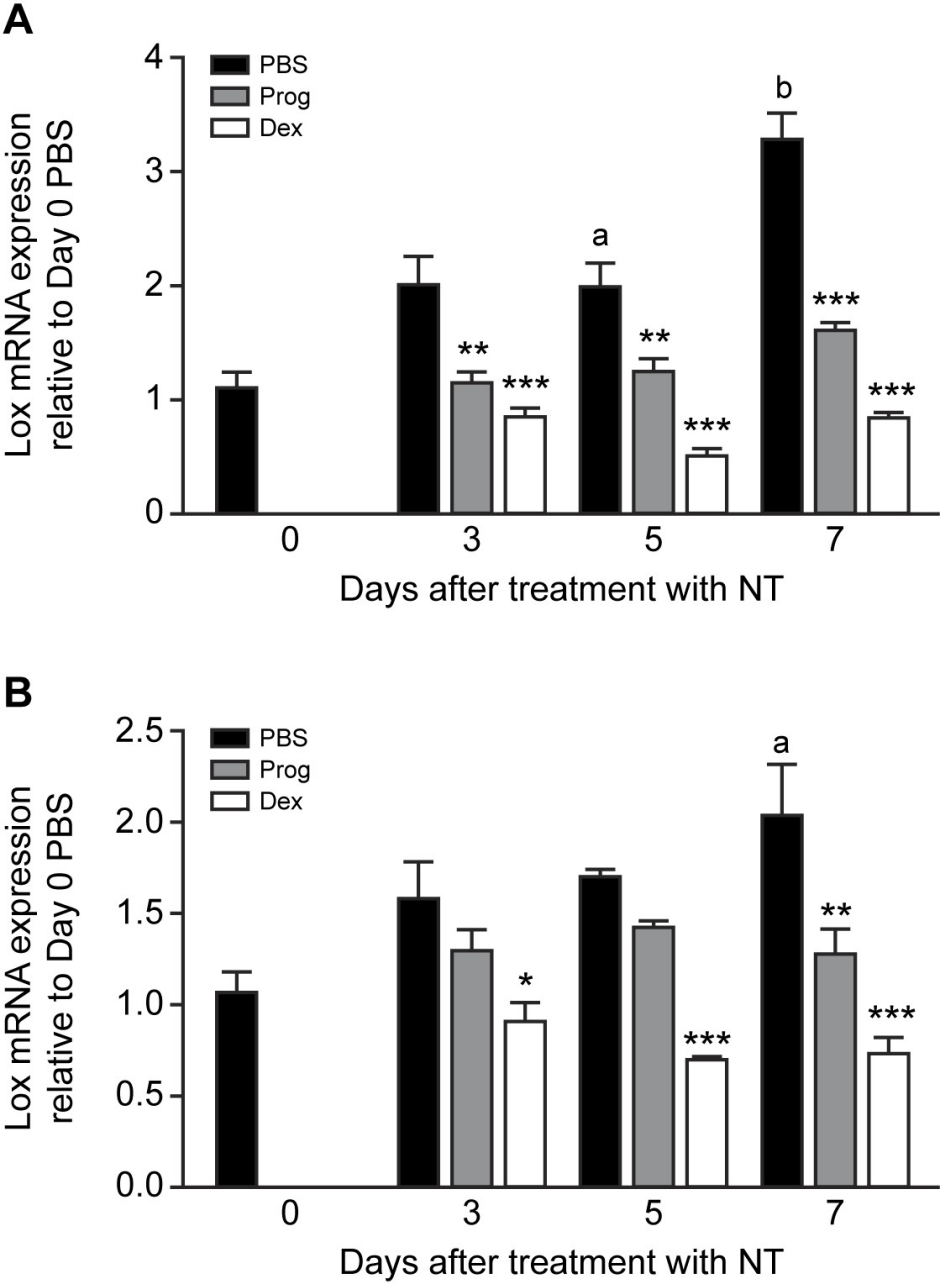
Time-dependent effects of progesterone and dexamethasone on NT-induced fibrotic gene expression in abdominal wall PMC. Effect of i.p treatment with carbon nanotube (NT, 25 μg) followed by daily injection for up to 7 days with PBS (vehicle), progesterone (Prog, 10 μg/kg) or dexamethasone (Dex, 1 μg/kg) on *Lox* (A) and *Col1a1* (B) mRNA expression by abdominal wall peritoneal mesothelial cells (PMC) *in vivo*. mRNA expression is shown relative to control (day 0 untreated animals) and is the mean ± SEM of 6–8 animals per treatment. a, p<0.05; b, p<0.01 compared with day 0 untreated animals (PBS). *, p<0.05; **, p<0.01; ***, p<0.001 compared with corresponding treatment with PBS. Two-way ANOVA with Bonferroni multiple comparison post-hoc testing.

Practical considerations meant that it was not possible to consistently collect mesothelial cell mRNA from the diaphragm. However, we were able to consistently collect mRNA from the PMC on a 6 cm^2^ section of abdominal wall. To determine if NT treatment induced fibrotic gene expression in these PMC cells, mRNA was extracted up to 7 days after treatment with NT. There was a time-dependent increase in both *Lox* and *Col1a1* mRNA expression (p<0.05) (Fig 4A and 4B). Furthermore, both progesterone (p<0.01) and dexamethasone treatment for 3, 5 and 7 days after NT injection (p<0.001) prevented the NT-induced rise in *Lox* mRNA (Fig 4A). A similar effect of progesterone on *Col1a1* mRNA (p<0.01 on Day 7) and dexamethasone (p<0.001 on Days 5 and 7) was observed (Fig 4B). At all time points dexamethasone was more effective than progesterone at inhibiting the effect of the NT.

### Effect of progesterone, dexamethasone and progesterone plus dexamethasone on NT-induced fibrotic gene expression in abdominal wall PMC

**Fig 5:**
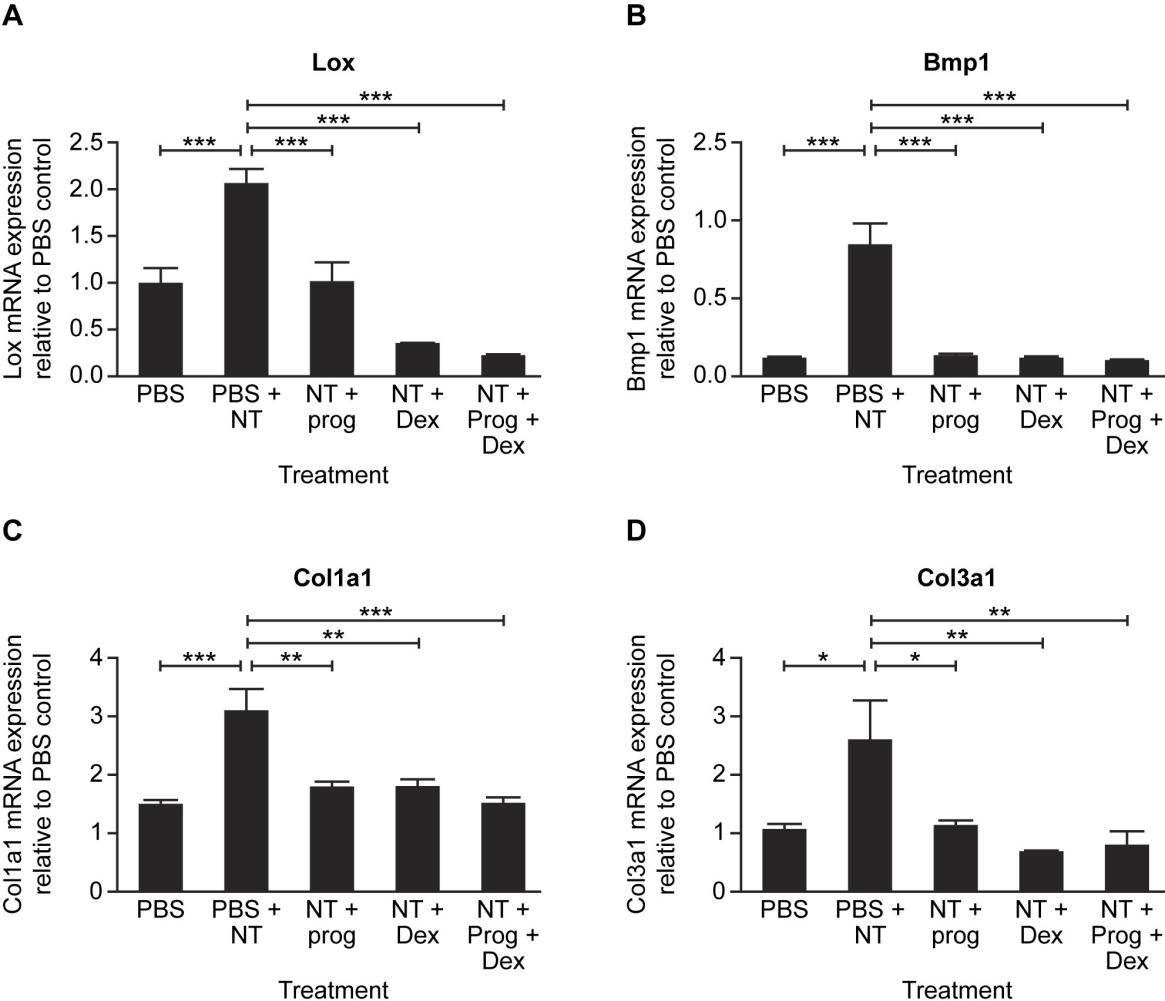
Progesterone and dexamethasone reduce NT-induced fibrotic gene expression in abdominal wall PMC. Effect of i.p treatment with carbon nanotube (NT, 25 μg) followed by daily injection for 7 days with PBS (vehicle), progesterone (Prog, 10 μg/kg), dexamethasone (Dex, 1 μg/kg) or Prog+Dex on *lox* (A), bone morphogenetic protein 1 (*Bmp1*) (B), *Col1a1* (C) and *Col3a1* (D) mRNA expression by abdominal wall peritoneal mesothelial cells *in vivo*. mRNA expression is shown relative to control day 0 untreated animals (PBS) and is the mean ± SEM of 8–10 animals per treatment. *, p<0.05; **, p<0.01; ***, p<0.001. One-way ANOVA with Tukey’s multiple comparison post-hoc testing.

Selecting the 7-day time-point after NT injection, we measured the effect of progesterone, dexamethasone and a combination of these steroids on the expression of *Lox*, the LOX and collagen processing enzyme *Bmp1*, and the collagens *Col1a1* and *Col3a1* mRNA. In all cases, NT injection significantly increased mRNA expression (p<0.05), whereas progesterone, dexamethasone or the two steroids combined consistently prevented this increase (p<0.05) (Fig 5). There was no evidence of an additive effect of progesterone and dexamethasone (Fig 5).

### Administration of lox miRNA lentiviral constructs reduces abdominal wall PMC lox mRNA expression in vitro and in vivo and reduces fibrosis on the diaphragm

**Fig 6:**
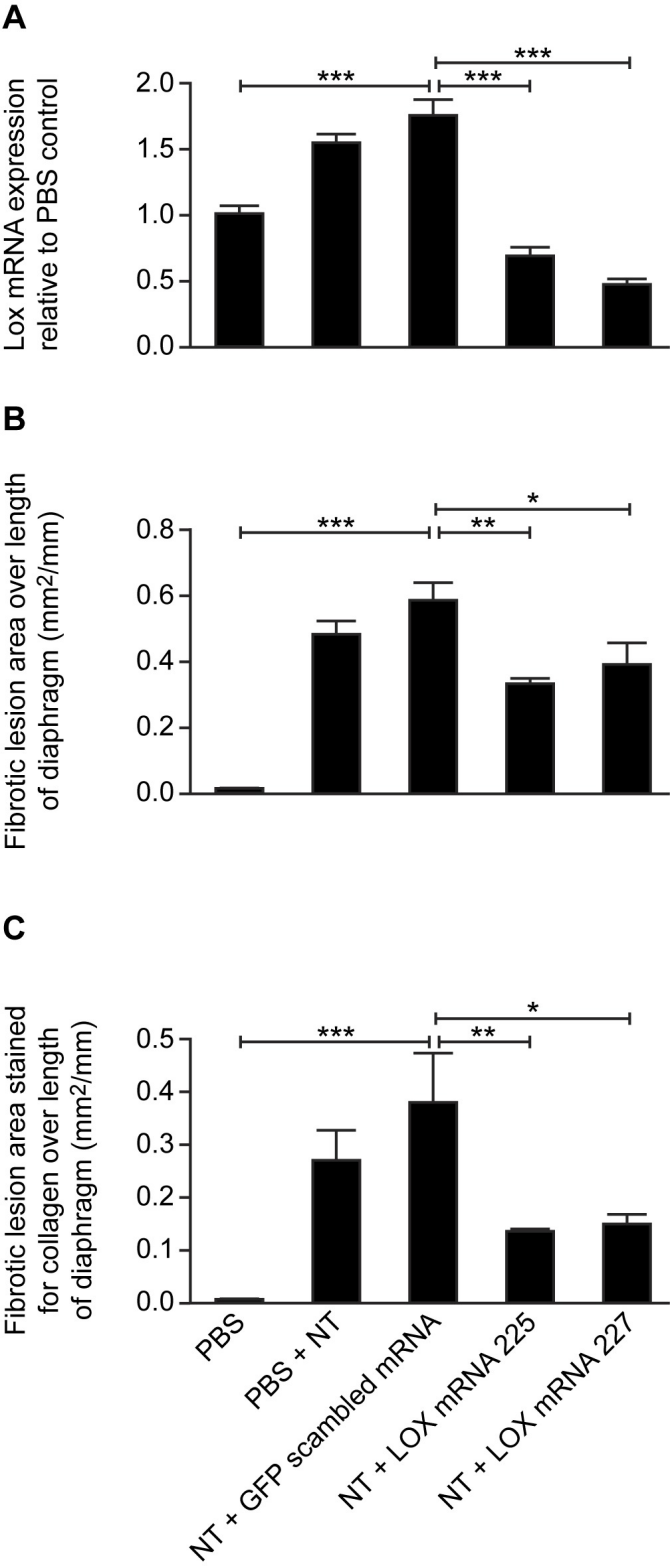
Effect of lentiviral lox miRNA construct on carbon nanotube (NT) induced fibrosis on the abdominal wall and diaphragm. Vehicle, scrambled miRNA and two selected *Lox* miRNA lentiviral constructs were injected i.p. followed 2 days later by 25 μg NT (except for the control group which received vehicle (PBS)). A), *Lox* mRNA in abdominal wall peritoneal mesothelial cells; B) fibrotic lesion area and C), extent of collagen staining within the lesion on the diaphragm. *, p<0.05; **, p<0.01; ***, p<0.001. One-way ANOVA with Tukey’s multiple comparison post-hoc testing.

To confirm that Lox expression is required for the development of fibrosis in response to NT treatment, we developed miRNA constructs that target *Lox* mRNA. Six different lentivral miRNA constructs transfected into mouse PMC *in vitro* inhibited *Lox* mRNA expression (p<0.001) (S1 Fig). Constructs L-225 and L-227, which target the *Lox* mRNA variants 1, 2 and 3 at nucleotides 1017 and 1205 bp respectively, but do not target any of the *Lox*-like mRNA sequences, inhibited *Lox* mRNA expression by >90% *in vitro*, were selected for *in vivo* use. Both constructs inhibited NT-induced *Lox* mRNA expression in PMC *in vivo*, compared to a scrambled miRNA sequence (p<0.001) (Fig 6A). Furthermore, both constructs also inhibited NT-induced fibrotic granuloma lesions (Fig 6B) and fibrotic granuloma lesion area stained for collagen (Fig 6C) (p<0.05)

### Progesterone ameliorates cytokine induced lox mRNA expression by peritoneal mesothelial cells in vitro

**Fig 7:**
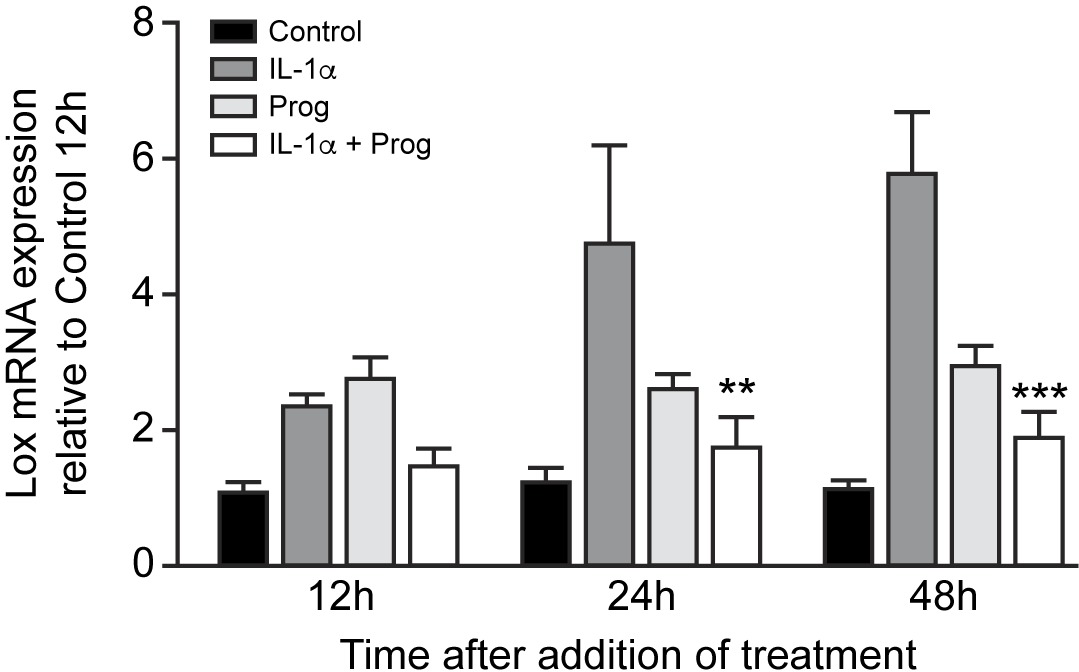
Time dependent effects of IL-1α (2.5 ng/ml and progesterone (Prog) (1 μM) on *Lox* mRNA expression by abdominal wall peritoneal mesothelial cells *in vitro*. mRNA expression is shown relative to control cultures at 12 h and is the mean ± SEM of 3 separate cultures using cells obtained from 6 mice. **, p<0.01; ***, p<0.01 compared to corresponding treatment with IL-1α alone. Two-way ANOVA with Bonferroni multiple comparison post-hoc testing.

To determine if mouse peritoneal mesothelial cells are responsive to physiological levels of progesterone, the effect of 1 μM progesterone on IL-1α induced *Lox* mRNA expression *in vitro* was measured for up to 48 h. After both 24 and 48 h, the IL-1α induced expression of *Lox* was significantly inhibited by 1μM progesterone (p<0.01 and p<0.001 respectively) (Fig 7). IL-1α alone induced *Lox* expression in a time-dependent manner, although this did not quite reach statistical significance (p = 0.057) (Fig 7).

## Discussion

We have developed a short-term mouse model of peritoneal fibrosis and abdominal wall fibrotic gene expression that implicates a key role of Lox in peritoneal fibrosis and adhesions. We have shown that the widely used Lox inhibitor, BAPN, as well as progesterone and dexamethasone, reduce NT-induced fibrosis and collagen deposition on the diaphragm. Furthermore, using a novel method to collect PMC mRNA we provide evidence that progesterone and dexamethasone act by blocking expression of NT-induced Lox and fibrotic genes in PMC, suggesting a mechanism for anti-fibrotic effects of steroids in the abdominal cavity.

The NT-induced fibrosis model [19] exhibited a rapid inflammatory response evidenced by accumulation of polymorphonuclear leukocytes and protein exudation after 24 h, followed by the formation of fibrotic granulomatous tissue on the peritoneal surface of the diaphragm, after 7 days. We observed a similar formation of granulomatous collagen-rich fibrotic plaques in our model, and a 60% inhibition of plaque formation and 90% reduction in collagen content by the irreversible inhibition of Lox enzyme activity by BAPN. These data suggest a key role of Lox in the fibrotic response to NT. Electron microscopy showed a sub-epithelial localization of collagen fibres, increased ordered collagen fibre deposition in response to NT, and disruption of fibre organization in response to BAPN, similar to the effect of BAPN on collagen lamellar stacking observed in the cornea [24], confirming a likely role of Lox-regulated collagen cross-linking in the fibrotic response. C57Bl/6 has previously been identified as a good choice of mouse strain in which to study peritoneal fibrosis, with this strain exhibiting a significant peritoneal wall thickening and *Col1a2* mRNA expression in response to i.p. adenovirus expressing TGFß1 [25].

The probable mechanism of NT-induced changes in the peritoneal mesothelium involves incomplete phagocytosis of these long NT by recruited macrophages leading to cytokine release, producing an amplified pro-inflammatory response and further cytokine release by PMC [26]. IL-1α increases *LOX* mRNA expression in human OSE (which are morphologically similar to PMC) *in vitro* [16,27], but the effect of cytokines on *LOX*/*Lox* expression has not been previously described in human or mouse PMC. Whilst we only occasionally observed adhesions in the form of liver to diaphragm anastomosis, and abdominal wall of NT treated mice did not show the regular presence of overt fibrotic plaques or NT deposits, the NT model has previously been shown to promote formation of intraperitoneal adhesions after 10 weeks or more of exposure [28]. NT are believed to accumulate on the abdominal surface of the diaphragm due to anteriorly directed transport [29,30], which explains the paucity of NT observed on the abdominal wall.

Macrophages are one of the major cell types contributing to the fibrotic granulomatous tissue on the peritoneal surface of the diaphragm after NT treatment [19 and the present study]. However, the precise role of macrophages in the formation of adhesions is unclear, and they have alternately been proposed to protect from or promote adhesion formation [31,32]. In a conditional macrophage ablation mouse model, depletion was associated with the appearance of abdominal adhesions, although the authors concede that the dimerizer used to induce the ablation may have caused injury to the mesothelial cells [33].

The mechanism of increased abdominal wall PMC fibrotic gene expression is unclear. However, we believe that the progressive increases in fibrotic gene expression in abdominal wall PMC observed in our study represent pre-fibrotic changes that herald the formation of discreet fibrotic lesions and adhesions in the abdominal cavity, presumably initiated by the NT-induced leukocytic accumulation [19] and release of inflammatory cytokines [28,34]. Indeed, evidence of a pre-fibrotic response in the abdominal wall PMC was apparent from elevations in *Lox*, *Col1a1* and *Col3a1* mRNA expression, with NT causing a progressive increase in abdominal wall *Lox* and *Col1a1* over 7 days. Furthermore, mRNA for *Bmp1 encoding* procollagen C-proteinase type 1 (responsible for both cleavage of the C-terminal propeptide region of procollagens type I, II and III, as well as for processing of pro-LOX protein to mature LOX enzyme [35]) is also increased after NT treatment. Our findings are consistent with the observation of increased *Col1a1* mRNA expression in mice treated intraperitoneally with chlorhexidine gluconate and glucose degradation products, which induce a fibrotic response in the peritoneum [17], and increase *COL3A1* mRNA, observed in an *in vitro* human primary PMC induced fibrosis study [36].

In addition to stimulating Lox and collagen formation, inflammatory mediators in the peritoneal cavity may act through alternative mechanisms to promote adhesions. Clinical and animal model studies indicate that mesothelial plasminogen activator (PA) activity is reduced after surgery or laparoscopy, due to reduced tissue (t)PA and increased PA inhibitor (I) [37,38]. Similarly, in a mouse model of post-inflammatory adhesion formation, CCL1 induced *Pai-1* and inhibited *Tpa* mRNA expression in peritoneal macrophages, as well as inducing *Pai-1* in PMC cells [39]. Together, this could lead to inhibition of the fibrinolytic pathways that accompany normal wound healing, and promotion of fibrosis.

To investigate if inhibition of *Lox* mRNA could reduce fibrosis on the diaphragm, we used *Lox* miRNA to knockdown levels of Lox. To our knowledge, this represents the first *in vivo Lox* knockdown in the peritoneal cavity. Reduction of NT-induced fibrosis and collagen deposition confirms the importance of PMC Lox synthesis in the development of the fibrotic response and the deposition of collagen.

Dexamethasone has known anti-inflammatory actions, decreasing vascular permeability and inhibiting cytokine and chemokine secretion. Animal models showed dexamethasone treatment leads to reduction of pneumoperitoneum-induced adhesion formation in mice [40,41], peritoneal adhesion formation in rats [42] and surgically-induced adhesion formation in rabbits [43], although the mechanism remains unclear. Evidence for improvement in adhesion scores by dexamethasone in randomized controlled clinical trials is conflicting [2]. We clearly demonstrate that dexamethasone reduces NT-induced expression of *Lox*, *Col1a1*, *Col3a1* and *Bmp1* mRNA in abdominal wall PMC *in vivo*. GR are present on human PMC cells, and cortisol also upregulates *11ßHSD1* mRNA (and potentially augments its own formation in a positive feedback loop)[44]. Whether or not this occurs by a direct effect on the PMC cells *in vivo* is unclear, but it could equally involve a reduction in macrophage-generated cytokines.

Progesterone has long been recognised to have anti-inflammatory and immunosuppressive effects, and was shown to reduce granuloma formation in a subcutaneous fibrosis model in rats [45]. Peritoneal adhesion formation in a guinea pig fibrosis model was significantly reduced by i.p. administration of 10 mg/kg progesterone [46]. We show that daily i.p. administration of similar doses of progesterone inhibit NT-induced expression of *Lox*, *Col1a1*, *Col3a1* and *Bmp1* mRNA in abdominal wall PMC *in vivo*.

Intriguingly, LOX activity in the mouse cervix (a collagen rich tissue that undergoes extensive remodeling) is lowest at dioestrus, and declines throughout pregnancy [47], inversely proportional to the levels of plasma progesterone, suggesting a possible causal relationship. Progesterone levels in follicular fluid can reach levels in the 10–100 μM range in women [48] and mice [49], and the high production rate of progesterone by the corpus luteum is reflected in progesterone levels 4–5 times higher in peritoneal fluid than in peripheral plasma [50].

Our observation that 1 μM progesterone suppresses IL1α-induced expression of *Lox* mRNA by mouse PMC by *in vitro*, suggests that the steroid may act directly on PMC to limit the fibrotic response to inflammatory signals. This concentration of progesterone is similar to the 175 nM concentration observed in human peritoneal fluid close to the ovary on postovulatory day 5 [50]. Furthermore, this suppression of IL-1α induced *Lox* is reminiscent of a similar reduction in cytokine-induced *LOX* mRNA initiated by cortisol in human OSE cells *in vitro* [16]. Further evidence that progesterone could act directly on PMC is demonstrated by the presence of PR in human PMC [51]. Interestingly, progesterone (1 μM) consistently had similar anti-inflammatory effects (increasing *11ßHSD1* and reducing *COX-2* mRNA) to cortisol on human PMC *in vitro*, albeit not significant [51]. Given that periovulatory follicular fluid progesterone can reach levels 10- to 100-fold higher than this [48], these data support the concept that progesterone could act to limit fibrosis in the PMC.

A local anti-inflammatory role of cortisol and/or progesterone has been proposed to provide protection from scarring to the ovarian surface after the wound-like event of ovulation [44,51]. Thus, locally high levels of both progesterone (in excess of 10 μM) and increases in both free and total cortisol in human follicular fluid after the LH surge [48] together with cytokine-driven increases in PR and GR expression, and 11ßHSD1 (which preferentially converts inactive cortisone to active cortisol) in OSE cells [44], provide a mechanism to limit inflammatory responses. The absence of such high levels of steroid in the vicinity of the abdominal PMC would explain their propensity to undergo fibrotic changes in response to injury. The anatomical and functional similarity of OSE to PMC would also explain how they might be able to respond to locally administered anti-inflammatory steroids. Whilst we would favour a direct action of progesterone and dexamethasone to attenuate *Lox* expression and subsequent fibrosis, our data do not unequivocally demonstrate such a mechanism. Global *Lox* knockout mice are embryonic lethal, [52], so further dissection of the mechanisms of these steroid effects would require a tissue specific conditional *Lox* knockout in mesothelial cells.

One of the hallmarks of inflammation in mesothelial cells is the onset of epithelial to mesenchymal transition (EMT) [25,53] and it is interesting to note in this context that, in a mouse model of liver fibrosis, PMC covering the liver undergo EMT and differentiate into hepatic stellate cells and myofibroblasts, leading to submesothelial “capsular” fibrosis”, associated with increased collagen deposition [54]. Whether this process involves increased expression of LOX is unknown, but this phenomenon likely implicates Lox in wider aspects of fibrotic disease.

In conclusion, our results provide proof-of-concept that intraperitoneal targeting of Lox may limit fibrosis and tissue adhesion. Lox-mediated suppression of collagen deposition is revealed as a key site of anti-inflammatory steroid action. Our findings have broad relevance to the use of anti-inflammatory agents to prevent adhesion formation following surgery or in association with other debilitating fibrotic conditions such as endometriosis and kidney dialysis.

## Supporting information

S1 Fig LOX mRNA expression in mouse peritoneal mesothelial cells transfected with LOX miRNA in vitro. Cells were treated for 16 h in serum-free medium containing scrambled miRNA or 6 different LOX miRNA sequences, followed by 24 h in serum containing medium. LOX mRNA expression is expressed relative to an untransfected control (No trans). Results are the mean±SEM of 3 separate cultures using cells obtained from 6 mice. ***p<0.001 compared with untransfected control.
(TIFF)

S2 Fig Collection method for mouse abdominal wall peritoneal mesothelial cell mRNA. A, The lateral wall of the abdominal wall pinned out on clean foil. The linea alba is visible near the left hand margin. B, Positioning of a 1cm deep section cut from a 50 ml Falcon tube over the exposed mesothelium. C, Addition of RNA lysis buffer. D) Scraping of the mesothelial surface with cell scraper. E, Removal of lysis buffer.
(TIF)

S3 Fig **Cytokeratin expression in mouse abdominal wall mesothelial cells without (A,C) and with (B,D) removal of mesothelial cells using lysis buffer and scraping.** Bar = 50 μm.
(TIFF)

S4 Fig Serial sections of a fibrotic granuloma lesion on the abdominal surface of the diaphragm, 7 days after carbon nanotube (NT) treatment. Sections are stained with picrosirius red (A), cytokeratin (B) and F4/80 (C). NT are clearly visible within the granuloma lesion, associated accumulations of macrophages (M). A partially intact mesothelial cell layer (PMC) is visible beneath the granuloma lesion. The granuloma lesion also contains a numerous tightly packed cells with large nuclei, presumed to be B lymphocytes (BL). Bar = 50 μm.
(TIF)

S5 Fig **Immunohistochemical localization of glucocorticoid receptor (A,C,E,G) and progesterone receptor (B,D,F,H) in ovarian surface epithelial cells (A,B), abdominal wall mesothelial cells (C,D) and diaphragm mesothelial cells (E,F).** Specific nuclear localisaton is indicated by the arrows. G,H, uterus positive control (insets are negative control tissue incubated without primary antibody). Bar = 50 μm (A,F) and 100 μm (G,H).
(TIF)

S6 Fig Fibrotic granuloma lesion on the peritoneal surface of the abdominal wall 7 days after carbon nanotube (NT) treatment, stained with picrosirius red. NT are clearly visible within the granuloma lesion. Bar = 50 μm.
(TIF)

S1 Table Lox RNAi target sequence and location of match in coding region of mouse LOX variants 1, 2 and 3 (NM_010728.3, 001286181.1 and 001286182.1), counting the start ATG as nucleotide 1. ^Basepairs (bp), *LOX-225 and 1396 were found to be identical and bold italic font indicates the loop sequences either side of each miRNA construct.
(PDF)

S2 Table Details of Taqman® gene expression assays-on-demand. (PDF)

S1 File Supplementary materials and methods. (PDF)
