# Supplementary figures and images for: Targeting lysyl oxidase reduces peritoneal fibrosis
Source: PLoS One. 2017 Aug 11;12(8):e0183013. doi: 10.1371/journal.pone.0183013 (PMC5553776; doi:10.1371/journal.pone.0183013)

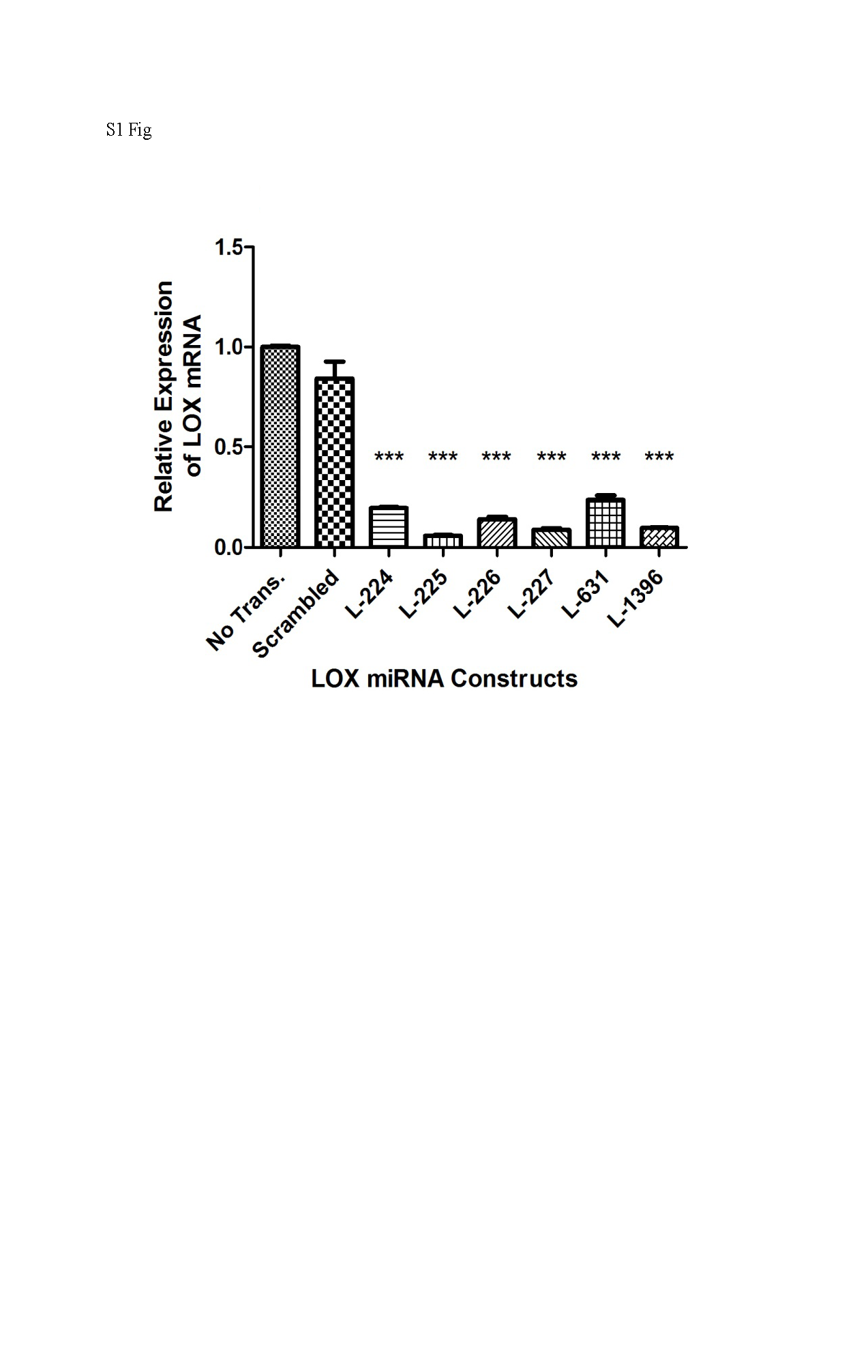

Supplement: S1 Fig — Cells were treated for 16 h in serum-free medium containing scrambled miRNA or 6 different LOX miRNA sequences, followed by 24 h in serum containing medium. LOX mRNA expression is expressed relative to an untransfected control (No trans). Results are the mean±SEM of 3 separate cultures using cells obtained from 6 mice. ***p<0.001 compared with untransfected control. (TIFF) [file pone.0183013.s001.tiff]

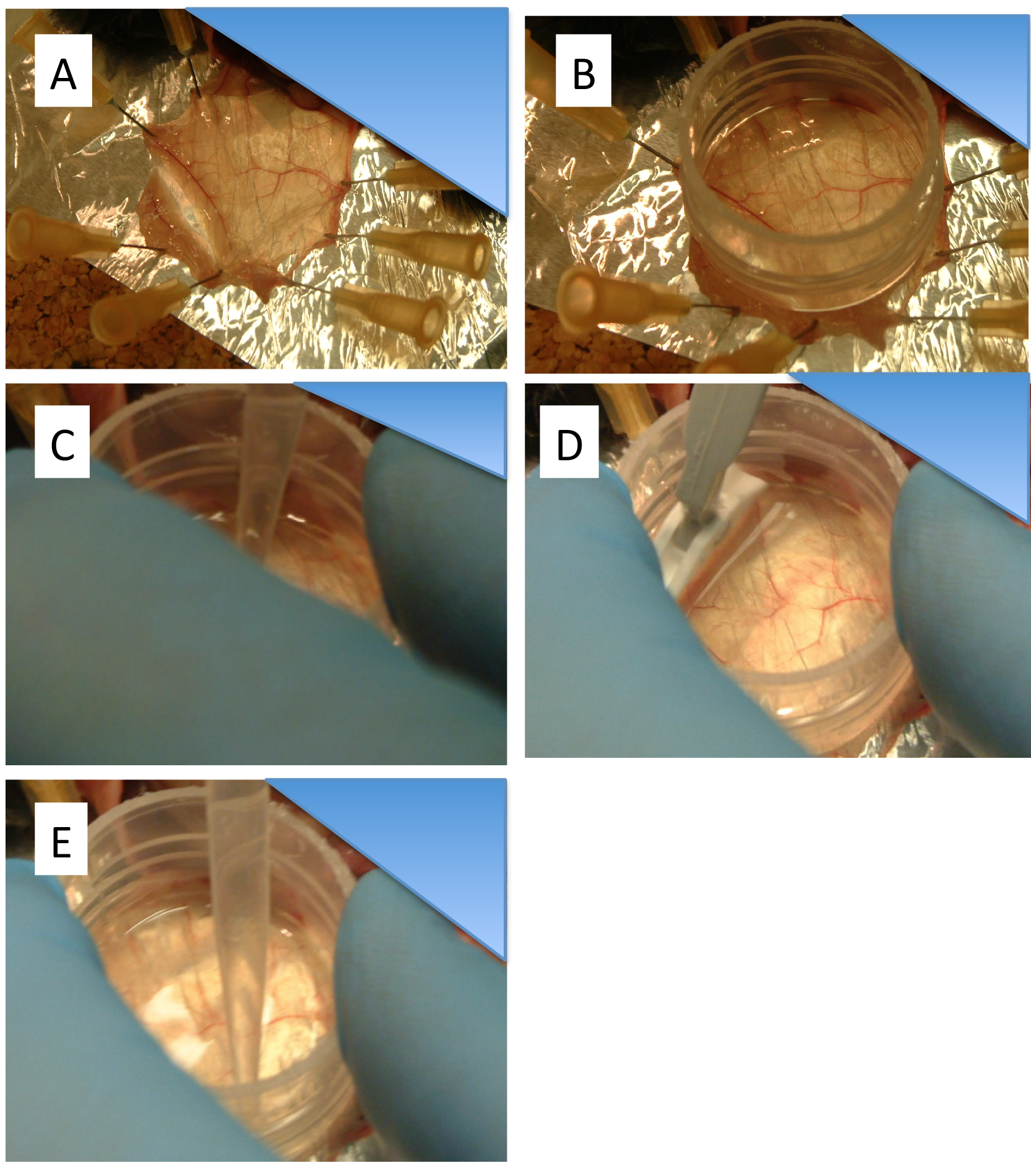

Supplement: S2 Fig — A, The lateral wall of the abdominal wall pinned out on clean foil. The linea alba is visible near the left hand margin. B, Positioning of a 1cm deep section cut from a 50 ml Falcon tube over the exposed mesothelium. C, Addition of RNA lysis buffer. D) Scraping of the mesothelial surface with cell scraper. E, Removal of lysis buffer. (TIF) [file pone.0183013.s002.tif]

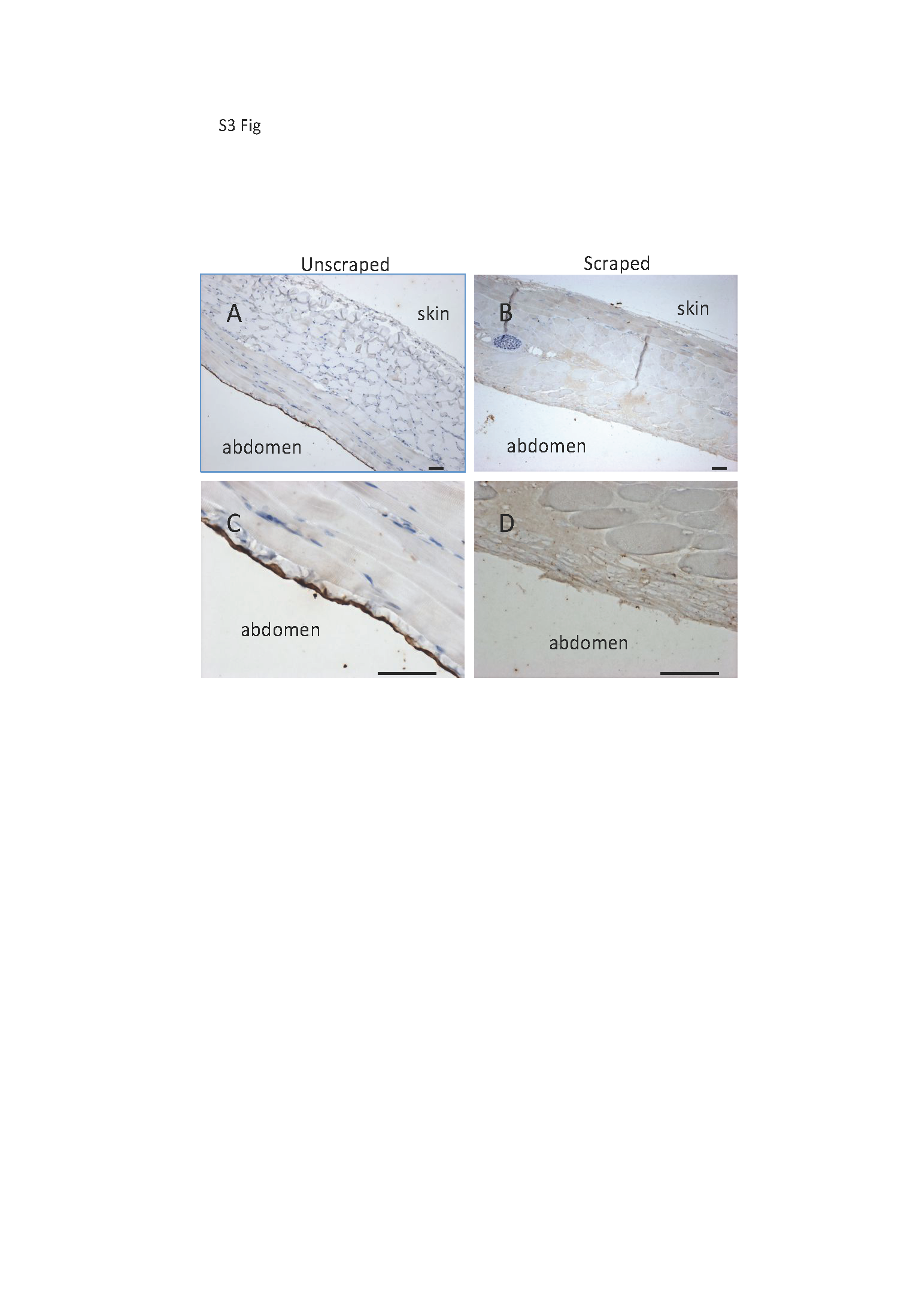

Supplement: S3 Fig — Cytokeratin expression in mouse abdominal wall mesothelial cells without (A,C) and with (B,D) removal of mesothelial cells using lysis buffer and scraping. Bar = 50 μm. (TIFF) [file pone.0183013.s003.tiff]

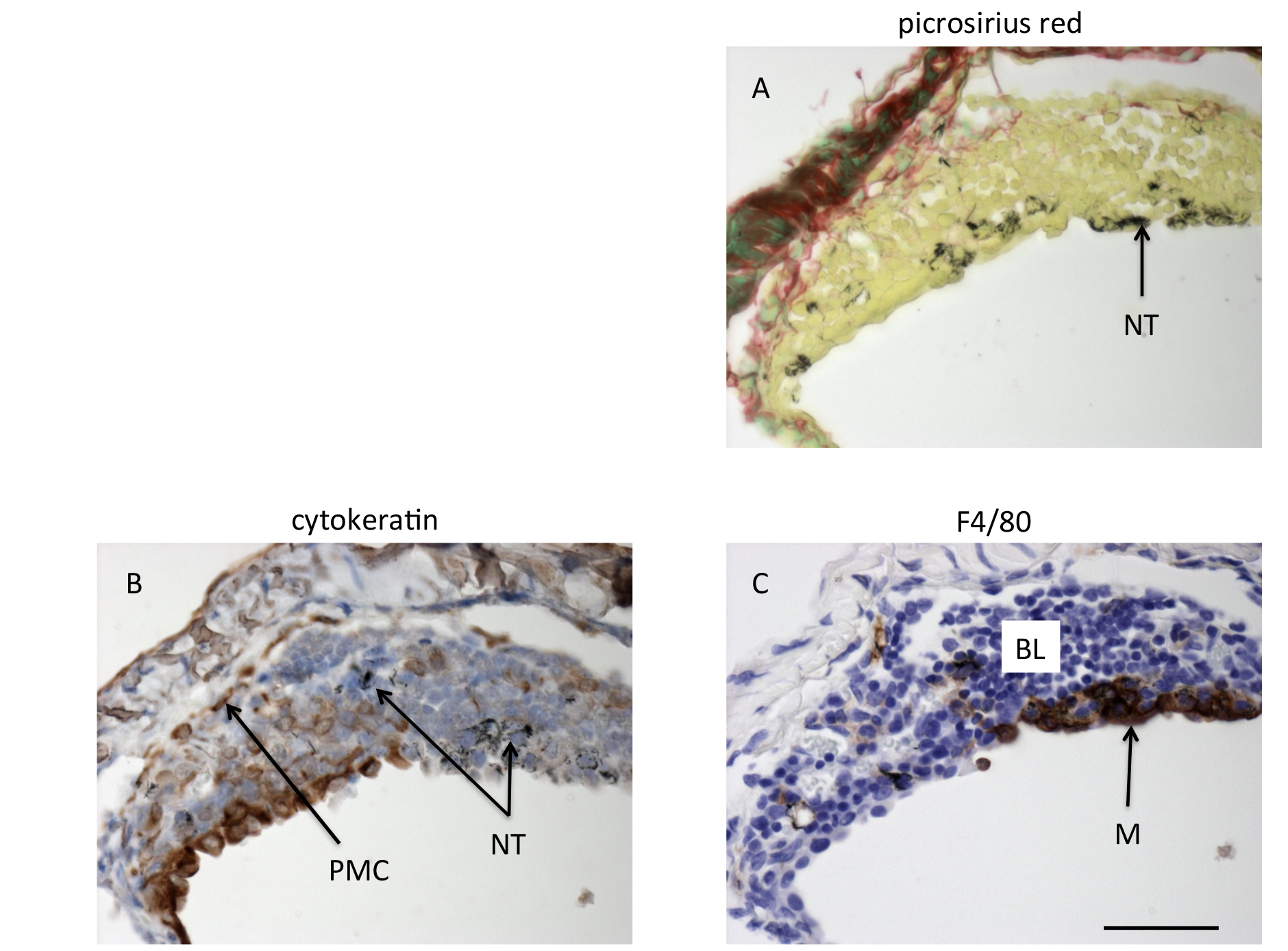

Supplement: S4 Fig — Sections are stained with picrosirius red (A), cytokeratin (B) and F4/80 (C). NT are clearly visible within the granuloma lesion, associated accumulations of macrophages (M). A partially intact mesothelial cell layer (PMC) is visible beneath the granuloma lesion. The granuloma lesion also contains a numerous tightly packed cells with large nuclei, presumed to be B lymphocytes (BL). Bar = 50 μm. (TIF) [file pone.0183013.s004.tif]

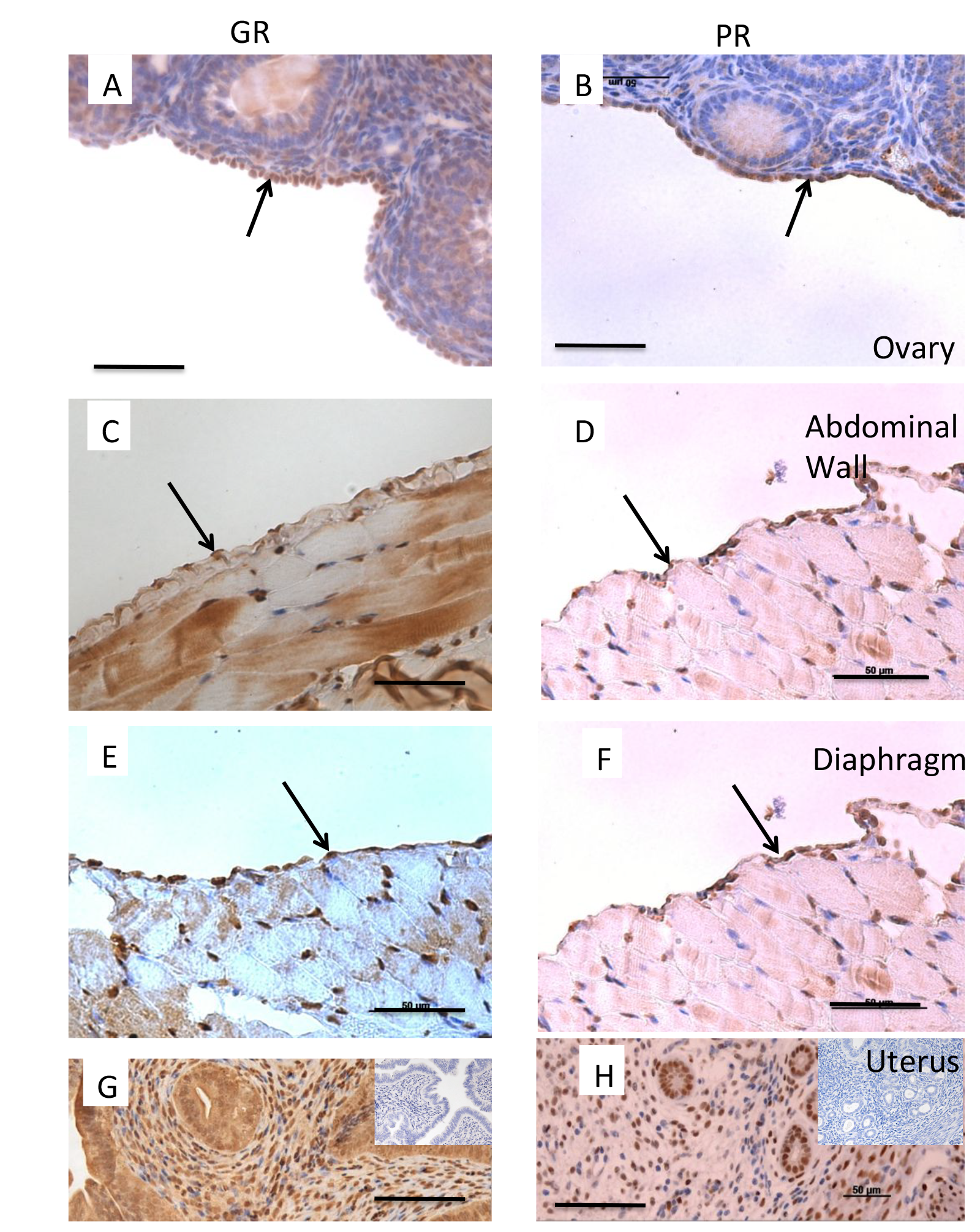

Supplement: S5 Fig — Immunohistochemical localization of glucocorticoid receptor (A,C,E,G) and progesterone receptor (B,D,F,H) in ovarian surface epithelial cells (A,B), abdominal wall mesothelial cells (C,D) and diaphragm mesothelial cells (E,F). Specific nuclear localisaton is indicated by the arrows. G,H, uterus positive control (insets are negative control tissue incubated without primary antibody). Bar = 50 μm (A,F) and 100 μm (G,H). (TIF) [file pone.0183013.s005.tif]

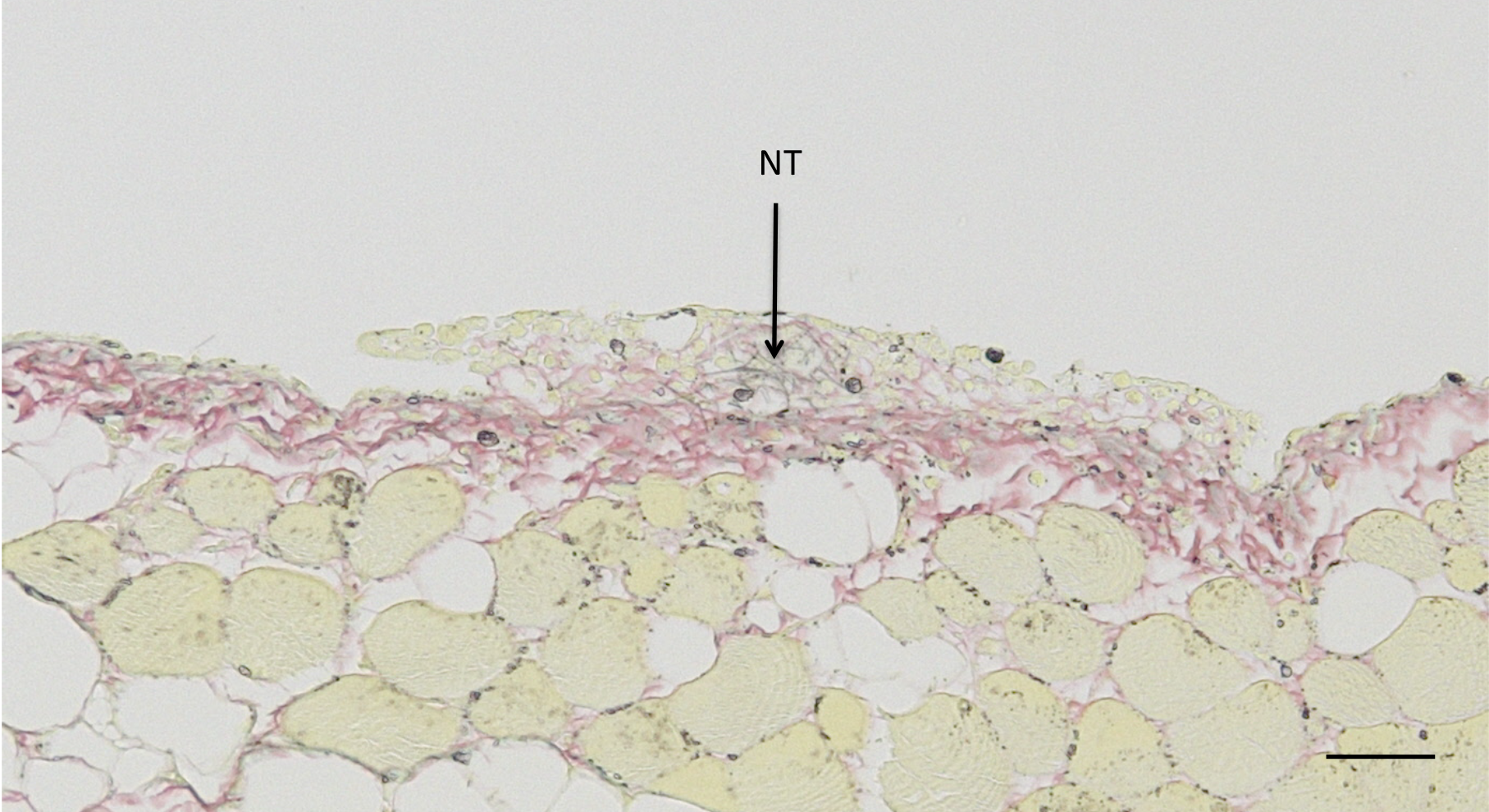

Supplement: S6 Fig — NT are clearly visible within the granuloma lesion. Bar = 50 μm. (TIF) [file pone.0183013.s006.tif]
