# Supplementary material for: Targeting lysyl oxidase reduces peritoneal fibrosis
Source: PLoS One. 2017 Aug 11;12(8):e0183013. doi: 10.1371/journal.pone.0183013 (PMC5553776; doi:10.1371/journal.pone.0183013)
Supplement: S1 Table — ^Basepairs (bp), *LOX-225 and 1396 were found to be identical and bold italic font indicates the loop sequences either side of each miRNA construct. (PDF) [file pone.0183013.s007.pdf]

S1 Table

**Construct designation**	**LOX target Sequence bp**	**Construct sequence (5'-3')**
Scrambled Control	NA	***TGC TG***A AAT GTA CTG CGC GTG GAG AC T***TT TGG CCA CTG ACT*** GAC GTC TCC ACG CAG TAC ATT T
LOX-224	663 bp^	***TGC TG***T ACA TAG ACA TCT TCT GGA CGG T***TT TGG CCA CTG AC***T GAC CGT CCA GAA TGT CTA TGT A
LOX-225*	1017 bp	***TGC TG***T AAC ATC CAG GAC TCA ATC CCG T***TT TGG CCA CTG ACT*** GAC GGG ATT GAC CTG GAT GTT A
LOX-226	249 bp	***TGC TG***T CAC GCA GCA GAA GAA TGG GCG T***TT TGG CCA CTG ACT*** GAC GCC CAT TCC TGC TGC CTG A
LOX-227	1205 bp	***TGC TG***A AAT TGT GCA GCC TGA GGC ATG T***TT TGG CCA CTG ACT*** GAC ATG CCT CAC TGC ACA ATT T
LOX-631	252 bp	***TGC TG***T TGT CAC GCA GCA GAA GAA TGG T***TT TGG CCA CTG ACT*** GAC CAT TCT TCC TGC GTG ACA A
LOX-1396*	1017 bp	***TGC TG***T AAC ATC CAG GAC TCA ATC CCG T***TT TGG CCA CTG ACT*** GAC GGG ATT GAC CTG GAT GTT A
