# Supplementary material for: Targeting lysyl oxidase reduces peritoneal fibrosis
Source: PLoS One. 2017 Aug 11;12(8):e0183013. doi: 10.1371/journal.pone.0183013 (PMC5553776; doi:10.1371/journal.pone.0183013)
Supplement: S2 Table — (PDF) [file pone.0183013.s008.pdf]

S2 Table

Gene name	Code	Interrogated sequence	Exon boundary	Assay location	Amplicon length (bp)
Lysyl oxidase	Mm01265612_m1	NM_001286181.1	6-7	1340	71
Col1a1	Mm00801666_g1	NM_007742.3	49-50	4071	89
Col3a1	Mm01254476_m1	NM_009930.2	50-51	4460	136
Procollagen c-proteinase	Mm00802220_m1	NM_009755.3	2-3	503	52
