# Supplementary material for: Targeting lysyl oxidase reduces peritoneal fibrosis
Source: PLoS One. 2017 Aug 11;12(8):e0183013. doi: 10.1371/journal.pone.0183013 (PMC5553776; doi:10.1371/journal.pone.0183013)
Supplement: S1 File — (PDF) [file pone.0183013.s009.pdf]

Supplementary material on the internet

The following supplementary material may be found in the online version of this article.

Appendix S1 Supplementary materials and methods

Tissue staining and immunohistochemistry.

Immunohistochemistry was performed using a Leica Bond max immuno-staining robot. The following dilutions of primary antibodies were used: polyclonal rabbit anti-mouse glucocorticoid receptor: (1: 100 Santa Cruz GR-M20), polyclonal rabbit anti-human progesterone receptor: (1:250 Sigma SAB4502184) and mouse monoclonal anti-human pan cytokeratin: (1:1000 Sigma C2562), rat monoclonal anti-mouse F4/80 (pan macrophage) (1:500 eBioscience 14-4801, Hatfield, UK). Following antigen retrieval using Novocastra pH6 retrieval buffer (Leica Biosystems, Newcastle, UK) in a decloaking chamber, all primary antibodies, except F4/80 which required a 10 minute trypsin digestion pretreatment (0.5 ng/ml in 1 mM Tris/EDTA buffer, 37 C) (Impress anti-rat (mouse absorbed) kit) were stained as follows. Rabbit primary antibodies were detected using a Leica Refine HRP based polymer system and visualized with DAB before counterstaining with Haematoxylin. F4/80 was detected using an anti Rat impress polymer (Vector labs. UK MP-7444), and cytokeratin was detected using a mouse on mouse polymer IHC kit (Abcam ab-127055, Cambridge, UK). All antibody incubations were 120 minutes with 15 minute polymer incubations. When IHC and haematoxylin counterstaining were complete, slides were removed from the robot, dehydrated in graded ethanols before clearing in xylene and coverslipping with Pertex (Cellpath, Newtown, Powys, UK).

Images were captured using an Olympus Provis microscope (Olympus Optical Co., London, UK,) equipped with Zeiss Axiovision software and a Zeiss Axiocam HRc digital camera.

Lentiviral miRNA constructs.

Oligonucleotides complimentary to mouse lox mRNA were obtained from (Invitrogen), details of DNA sequences are shown in Supplementary table 1 (S1_Table), these were inserted into pcDNA6.2-GW_GFP (Invitrogen, Life Technologies, Paisley, Renfrewshire) as described [1] and shuttled into pLenti6.2_cppt_emGFP to create pLenti6.2_cppt_emGFP_miR-neg, -224, -225, -226, -227, -631 and -1396 as detailed in [2]. Transfection efficiency *in vitro* was assessed using mouse peritoneal mesothelial cells (see below).

Mesothelial cell culture

Mesothelial cells were collected and cultured as described previously (Calabro et al., 2009). In brief, sections of abdominal wall approximately 1cm^2 were isolated from six mice under aseptic conditions. The pieces of abdominal wall were incubated in 10 ml 0.25% trypsin/0.02% EDTA solution (Invitrogen) at 37 °C for 30 minutes, with gentle agitation. Trypsin was neutralized with 10 ml culture medium (Modified from [3]) (Phenol-red free RPMI (Invitrogen)with 15% Fetal calf serum (Sigma), 2 mM L-glutamine and ITS (Invitrogen), 20 mM HEPES and 10 μM 2-mercaptoethanol (Sigma), trace elements B (Cellgro), 50 IU/ml Penicillin and 50 μg/ml Streptomycin Sigma) and 500 ng/ml epidermal growth factor (Sigma) and mesothelial cells separated by centrifugation at 800 x*g*. Cells were resuspended in fresh culture medium and plated out into T75 tissue culture flasks (Corning). When 80-90% confluent, cells were trypsinised (0.05% trypsin/EDTA, Invitrogen).

For *in vitro lox* miRNA experiments, cells were plated out at 50,000 cells/well into 24-well tissue culture plates (Corning), pre-coated with bovine fibronectin (2.5 μg/ml in 0.5 ml PBS, Sigma). When 80-90% confluent, the cells were serum-starved and 6 LOX miRNA constructs, a scrambled sequence control and a no-transfection control were prepared in serum-free medium containing 0.1% polybrene (Sigma). Treatments were in triplicate overnight, after which serum-containing medium was substituted for a further 24 hours. Fluorescence imaging was then used to determine transfection success by means of nuclear GFP expression. Cells were lysed with 0.35 ml RNEasy lysis buffer and the lysate stored at -80C until required for RNA extraction.

For *in vitro lox* mRNA expression experiments, cells were plated out at 100,000 cells/well in triplicate into 24-well tissue culture plates (Corning). After 24 h, medium was replaced with serum-free medium containing 0.01% BSA (Fraction V, Sigma) for a further 24 h, after which it was replaced with control medium, or medium containing progesterone (1 μM, Sigma) or recombinant human IL-1α (2.5 ng/ml, R & D Systems Europe Ltd, Abingdon, UK) alone or in combination. Cultures were continued for 12, 24 and 48 h, after which the medium was removed and cell monolayers lysed with 0.35 ml RNEasy lysis buffer, and the lysate stored at -80 °C until required for RNA extraction.

References

1. Evans J, Catalano RD, Brown P, Sherwin R, Critchley HO, Faziebas AT, et al. Prokineticin 1 mediates fetal-maternal dialogue regulating endometrial leukemia inhibitory factor. FASEB J. 2009;23: 2165-2175.
2. Calabrò ML, Gasperini P, Di Gangi IM, Indraccolo S, Barbierato M, Amadori A, et al. Antineoplastic activity of lentiviral vectors expressing interferon-α in a preclinical model of primary effusion lymphoma. Blood. 2009;113: 4525–4533.

3. Bot J, Whitaker D, Vivian J, Lake R, Yao V, McCauley R. Culturing mouse peritoneal mesothelial cells. Pathol Res Pract. 2003;199: 341-344.

Supplementary figure legends

S1 Fig

Lox mRNA expression in mouse peritoneal mesothelial cells transfected with *Lox* miRNA in vitro. Cells were treated for 16 h in serum-free medium containing scrambled miRNA or 6 different *Lox* miRNA sequences, followed by 24 h in serum containing medium. LOX mRNA expression is expressed relative to an untransfected control (No trans). Results are the mean±SEM of 3 separate cultures using cells obtained from 6 mice. ***$p<0.001$ compared with untransfected control.

S2 Fig

Collection method for mouse abdominal wall peritoneal mesothelial cell mRNA. A, The lateral wall of the abdominal wall pinned out on clean foil. The linea alba is visible near the left hand margin. B, Positioning of a 1cm deep section cut from a 50 ml Falcon tube over the exposed mesothelium. C, Addition of RNA lysis buffer. D) Scraping of the mesothelial surface with cell scraper. E, Removal of lysis buffer.

S3 Fig

Cytokeratin expression in mouse abdominal wall mesothelial cells without (A,C) and with (B,D) removal of mesothelial cells using lysis buffer and scraping. Bar = 50 µm

S4 Fig

Serial sections of a fibrotic granuloma lesion on the abdominal surface of the diaphragm, 7 days after carbon nanotube (NT) treatment. Sections are stained with picrosirius red (A), cytokeratin (B) and F4/80 (C). NT are clearly visible within the granuloma lesion, associated accumulations of macrophages (M). A partially intact mesothelial cell layer (PMC) is visible beneath the granuloma lesion. The granuloma lesion also contains a numerous tightly packed cells with large nuclei, presumed to be B lymphocytes (BL). Bar = 50 µm.

S5 Fig

Immunohistochemical localization of glucocorticoid receptor (A,C,E,G) and progesterone receptor (B,D,F,H) in ovarian surface epithelial cells (A,B), abdominal wall mesothelial cells (C,D) and diaphragm mesothelial cells (E,F). Specific nuclear localisaton is indicated by the arrows. G,H, uterus positive control (insets are negative control tissue incubated without primary antibody). Bar = 50 µm (A,F) and 100 µm (G,H).

S6 Fig

Fibrotic granuloma lesion on the peritoneal surface of the abdominal wall 7 days after carbon nanotube (NT) treatment, stained with picrosirius red. NT are clearly visible within the granuloma lesion. Bar = 50 µm.
